# The co-infection of pulmonary hydatid cyst, lophomoniasis and tuberculosis in a patient with resistant respiratory symptoms; a case report study

**DOI:** 10.1186/s12879-023-08907-4

**Published:** 2024-01-02

**Authors:** Mohammad Hadi Tajik Jalayeri, Rahmat Allah Sharifi far, Narges Lashkarbolouk, Mahdi Mazandarani

**Affiliations:** 1 Pulmonary and Critical Care Division, Sayyad Shirazi Medical and Educational Center, Golestan University of Medical Sciences, Gorgan, Iran; 2 Infectious Diseases Research Center, Golestan University of Medical Sciences, Gorgan, Iran; 3 Golestan University of Medical Sciences, Gorgan, Iran; 4 Endocrinology and Metabolism Research Institute, Tehran University of Medical Sciences, Tehran, Iran

**Keywords:** Tuberculosis, Pulmonary hydatid cyst, Lophompnas, Parasite, Coinfection

## Abstract

**Background:**

*Lophomonas blattarum* is a rare protozoan that causes opportunistic infections, and the co-infection of lophomonas with tuberculosis and human hydatidosis is a serious public problem in the co-endemic areas of developing countries.

**Case report:**

We presented a 58-year-old female with fever, losing weight, and cough with whitish-yellow sputum that started one month ago. Increasing inflammatory markers and hypereosinophilia in laboratory tests, and a cavity with thick, regular walls and undulating air-fluid levels measuring 43 × 30, evident in the upper segment of the right lower lobe (RLL), along with consolidation and the ground glass opacity of the upper segment and posterior basal of the RLL is apparent in CT scan were reported. Then, a bronchoscopy was requested, and the BAL specimen reported a negative fungal and bacterial infection in the samples. Several live and oval flagellated lophomonas protozoa, hydatid cyst protoscoleces (the larval forms of the parasites), and *M. tuberculosis* were observed in microscopic evaluation. The patient was treated with metronidazole, oral albendazole, and a combination of TB regimen.

**Conclusion:**

Physicians should always consider the possibility of co-infections of lophomonas with tuberculosis and human hydatidosis and investigate patients with risk factors such as immunodeficiency conditions or treated with immunosuppressive medications.

## Introduction

*Lophomonas blattarum* (*L. blattarum*) is a rare protozoan parasite found in the intestines of some special arthropods, such as termites and cockroaches. *L. blattarum* is an opportunistic pathogen that causes bronchopulmonary infections, especially in immunocompromised patients This host-specific protozoan could be spread by waste and dust during the crawling of a host. It can infect various tissues, such as the sinuses and human reproductive and respiratory systems. Clinical manifestations are predominantly non-specific and, in most cases, include cough, sputum expectoration, fever, chest stiffness, and shortness of breath. Radiography findings may show signs of pneumonia, bronchiectasis, pulmonary abscess, and pleural effusion. Microscopic examination of respiratory secretions is the gold standard for diagnosing *L. blattarum* because this protozoan has similar symptoms to other infections. Therefore, bronchoscopy brush smears, biopsy smears, or bronchoalveolar lavage (BAL) can lead to a diagnosis. Metronidazole or tinidazole is usually prescribed in the treatment regimen for infected patients [1–7, 8].

Hydatidosis (*cystic echinococcosis* (CE)) is a zoonotic parasitic infection. The larval stages of *Echinococcus granulosus* (*E. granulosus*) develop in human internal organs and induce a granulomatous reaction in the host, followed by the development of a fibrous tissue layer. Hydatidosis is endemic in sheep and cattle breeding areas around the world. This parasite has a worldwide geographical distribution and is present in all countries. According to a World Health Organization (WHO) report, about one million people are infected with *E. granulosus* infection annually worldwide. Clinical findings depend on the organs’ involvement and the extent of infection, but no obvious clinical symptoms are often observed. Respiratory distress, cough, dyspnea, hemoptysis, hydatoptysis, and chest pain are usually reported in pulmonary *E. granulosus* infection. The radiology findings show homogeneous, round or oval, well-circumscribed lesions, cystic lesions, and consolidation. Diagnosis of *E. granulosus* infection is based on imaging tests, examination of cyst fluid, or serological tests (immunodiagnostic tests). The asymptomatic nature of the disease makes diagnosis difficult and increases the risk of transmission. Treating pulmonary *E. granulosus* infection could be pharmacotherapy or surgical intervention, and surgical intervention is the treatment choice. A benzimidazole group (albendazole and mebendazole) could be prescribed for medical treatment [4, 9–11].

*Mycobacterium tuberculosis* (*M. tuberculosis*) is an important infectious disease and one of the leading causes of morbidity and mortality worldwide. According to a 2019 WHO report, the incidence of tuberculosis in Iran is less than 10 cases per 100,000 subjects. *M. tuberculosis* can involve multiple organ systems, and timely diagnosis is vital because delayed treatment is associated with severe morbidity and mortality. Infectious *M. tuberculosis* is a respiratory disease associated with cough, sputum production, sweating, weight loss, weakness, malaise, and hemoptysis, which shows the importance of differentiating this disease from other respiratory infections. The gold standard for diagnosing active *M. tuberculosis* is a culture of *M. tuberculosis* from tissues or fluids of the affected area. The *M. tuberculosis* treatment program is known as direct observation of treatment by public health workers (DOTS) and with combination therapeutic regimens (isoniazid, rifampin, pyrazinamide, and either ethambutol or streptomycin) [1, 12–14].

To date, several cases of co-infection with lophomonas and other infectious diseases have been reported. Nevertheless, there is no evidence from around the world of this co-infection. In this case study, we describe the diagnosis and treatment of a 58-year-old woman infected with lophomonas, *E. granulosus*, and *M. tuberculosis* simultaneously.

## Case report

We admitted a 58-year-old female patient to the Department of Respiratory Medicine at Sayyad Shirazi Hospital, Gorgan, Iran. The patient complained of fever, chills, and cough with whitish-yellow sputum that started about one month ago. She had a history of losing significant weight during one month. Her symptoms gradually progressed in the last week, and her symptoms did not completely heal to outpatient treatment. On admission, the patient mentioned weakness, lethargy, and frequent fatigue for a month and had one episode of hemoptysis before admission; she coughed up a clot with ten cubic centimetre (cc).

She had a history of diabetes, a brain tumor, and hypothyroidism. The patient had brain surgery ten years ago and responded to surgical treatment. She is being treated for diabetes and hypothyroidism, which are under control. In physical examination, the patient’s vital signs were stable, and the oxygen saturation level was 96% without supplemental oxygen therapy. The respiratory crackle in the lungs was detected by auscultation, and heart sounds (S1 and S2) had a regular pattern. Surgical scars were visible on examination. The abdominopelvic examination was normal, and splenomegaly and hepatomegaly were not found.

Because the symptoms of the infection have responded and have always improved with outpatient treatment, the patient has not been fully evaluated for the cause of the recurrence of the respiratory infection. However, she was hospitalized due to the persistence of symptoms and abnormal examination findings. For primary evaluation, laboratory tests, chest X-rays requested, and X-rays findings showed pulmonary cavity with patchy consolidation in right lobe. On laboratory tests, the hemogram revealed anemia and leukocytosis with hypereosinophilia. The erythrocyte sedimentation rate (ESR) was 26 mm/s, and a C-reactive protein test (CRP) concentration was reported as + 1.

A consultation with a pulmonologist and infection specialist was requested. Based on increasing of inflammatory markers, hypereosinophilia and abnormal X-rays findings, additional laboratory test with spiral CT scan performed.

The serum levels of perinuclear antineutrophil cytoplasmic antibodies (p-ANCA), antineutrophil cytoplasmic autoantibody, cytoplasmic (c-ANCA), and viral markers (HIV, hepatitis C, and hepatitis B) were normal. The result of galactomannan index was 2.98 (positive > 1). In addition, because of the COVID-19 pandemic, the SARS-CoV-2 polymerase chain reaction (PCR) was done and came negative.

**Fig. 1:**
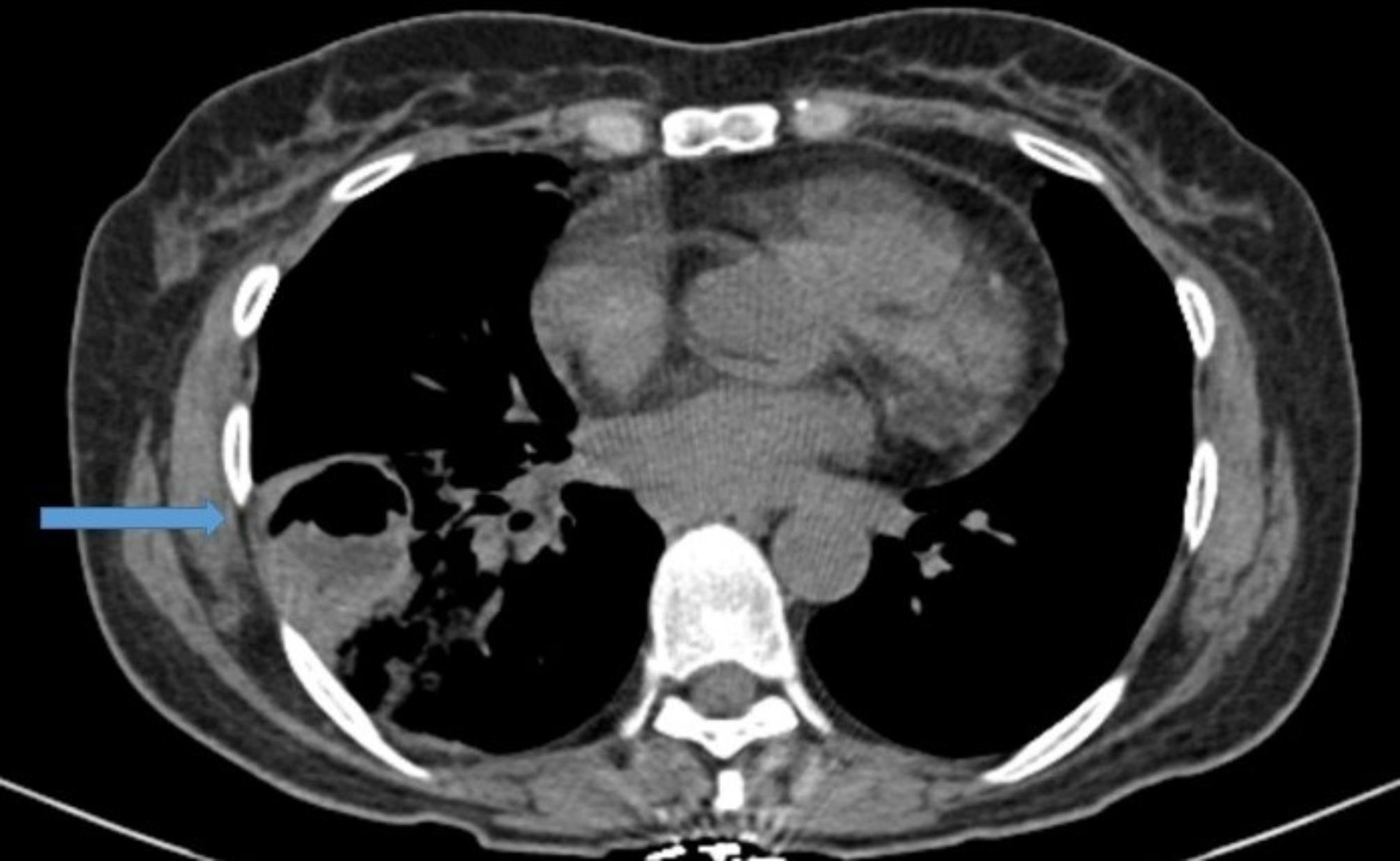
Mediastibal view of lung CT scan shows a cavity with thick, regular walls and undulating air-fluid levels measuring 43 × 30, evident in the upper segment of the RLL (blue arrow)

**Fig. 2:**
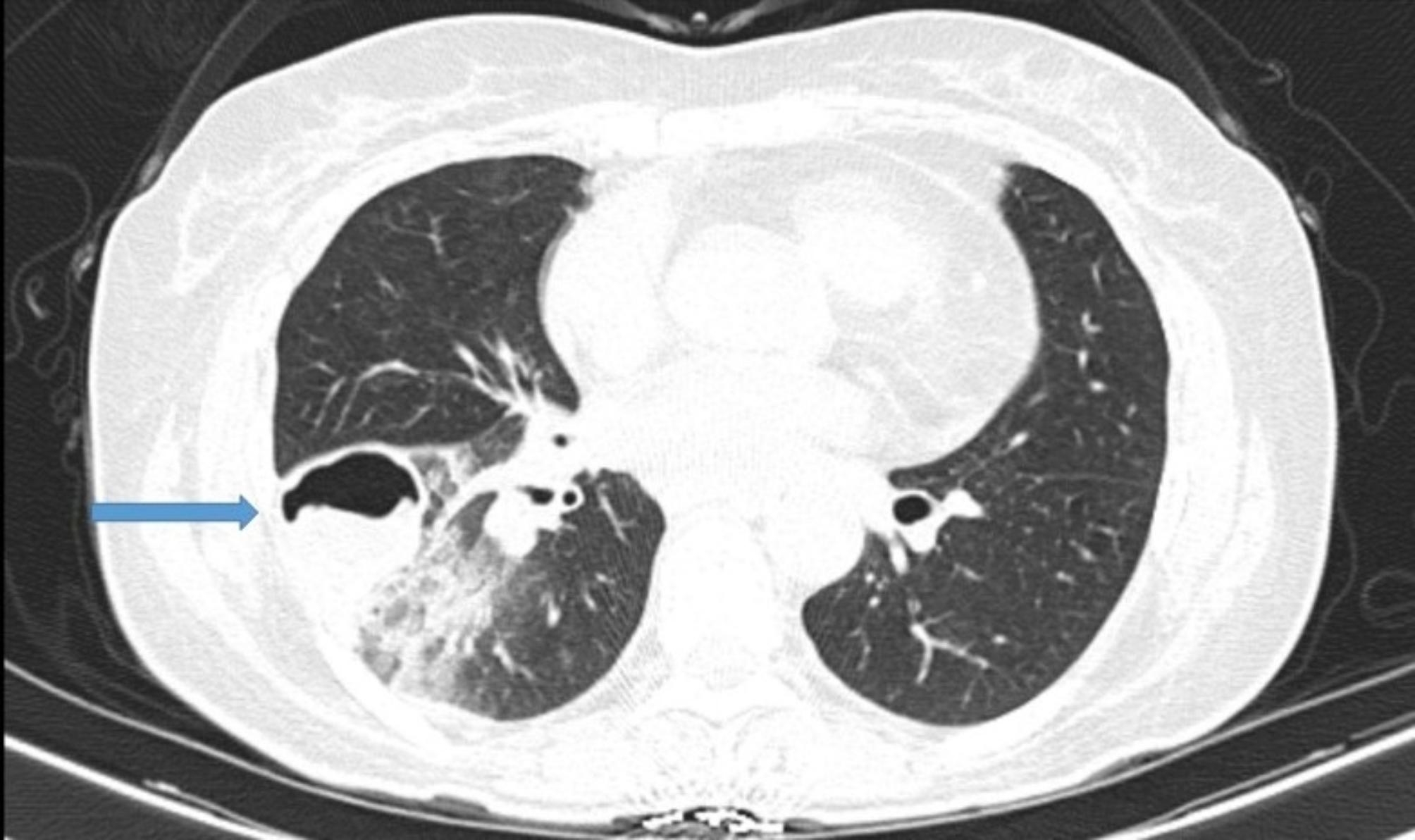
Lung CT scan shows a cavity with thick, regular walls and undulating air-fluid levels measuring 43 × 30, evident in the upper segment of the RLL (blue arrow)

**Fig. 3:**
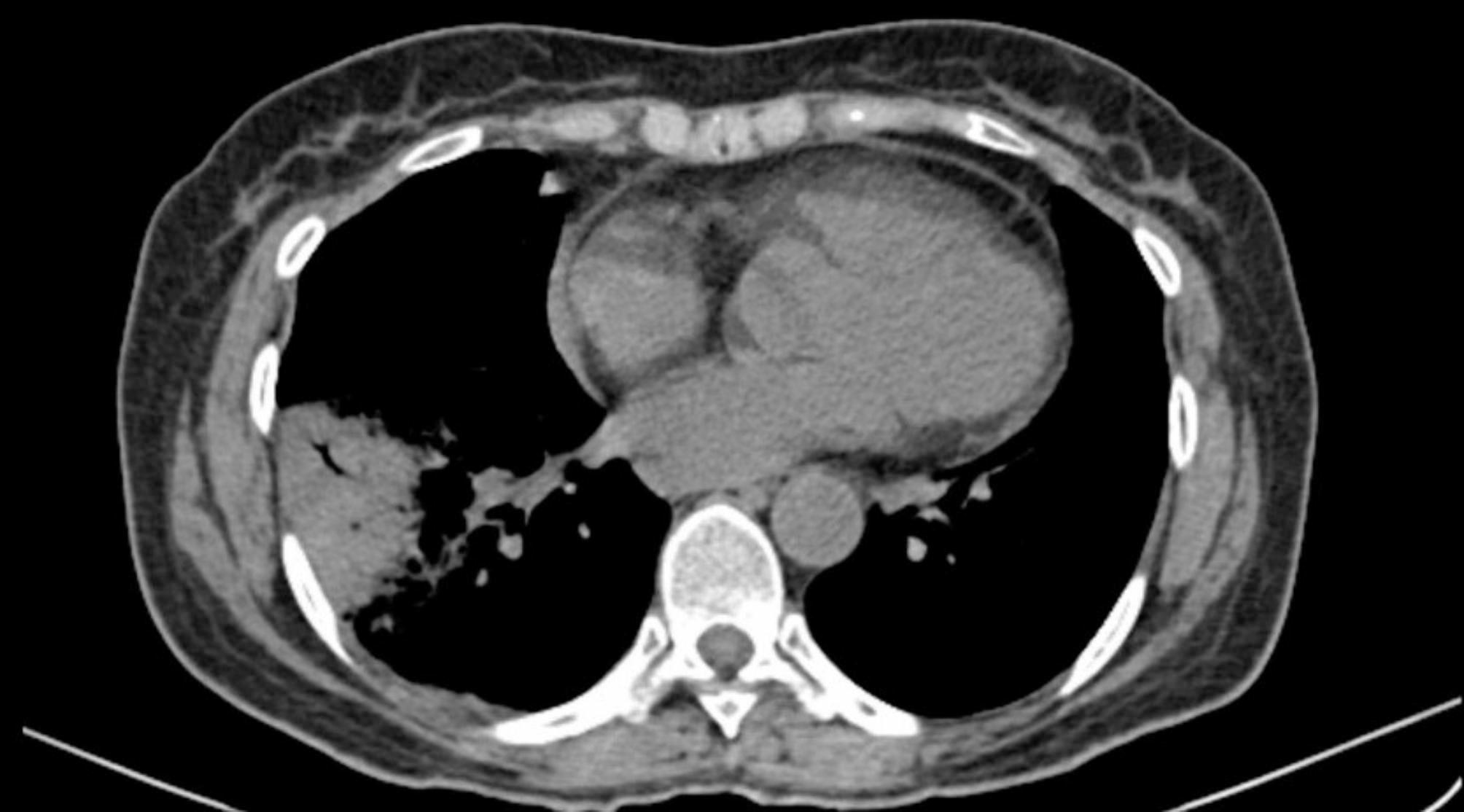
Mediastinal view of lung CT scan shows consolidation and opacity of the Grand Glass of the upper segment and posterior basal of the Right lower lobe

**Fig. 4:**
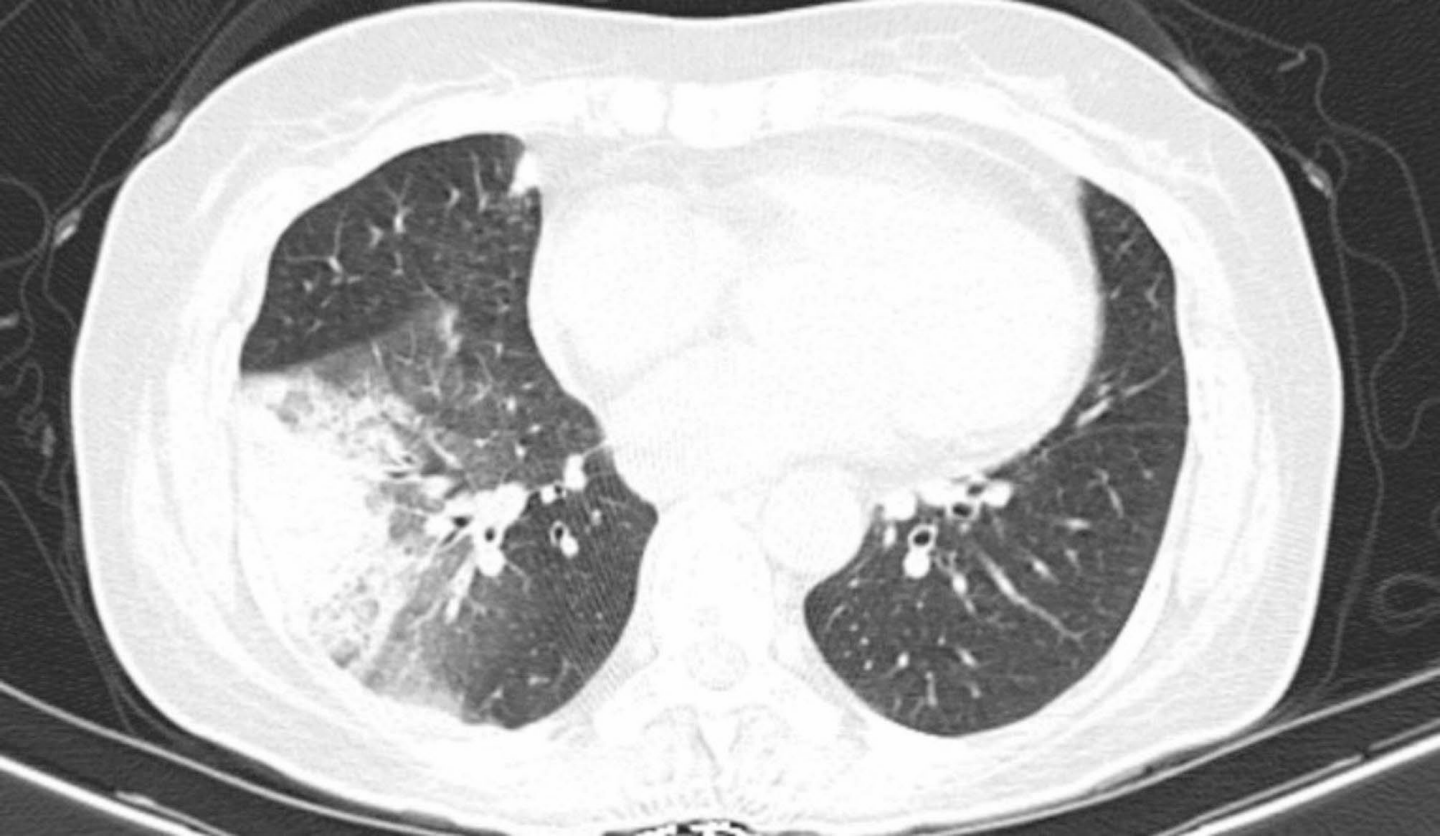
Lung CT scan shows consolidation and opacity of the Grand Glass of the upper segment and posterior basal of the Right lower lobe. Mild plural effusion is seen

The spiral CT scan of the lungs showed a cavity with thick, regular walls and undulating air-fluid levels measuring 43 × 30, evident in the upper segment of the right lower lobe (RLL). The consolidation and ground glass opacity of the upper segment and posterior basal of the RLL are apparent. The patient had a mild pleural effusion (approximately 100 cc) (Figs. 1, 2, 3 and 4).

Because of the abnormal lung CT scan, we ordered a serological test of *E. granulosus*, and a fiber optic bronchoscopy for the patient. Three samples of sputum were requested for culture. A BAL specimen was taken to evaluate BK (Bacillus of Koch), lophomonas, bacterial and fungal infections. *M. tuberculosis* infection was detected in sputum and BAL samples. We confirmed the diagnosis using the GeneXpert MTB/RIF PCR method, which also showed positive results.

**Fig. 5:**
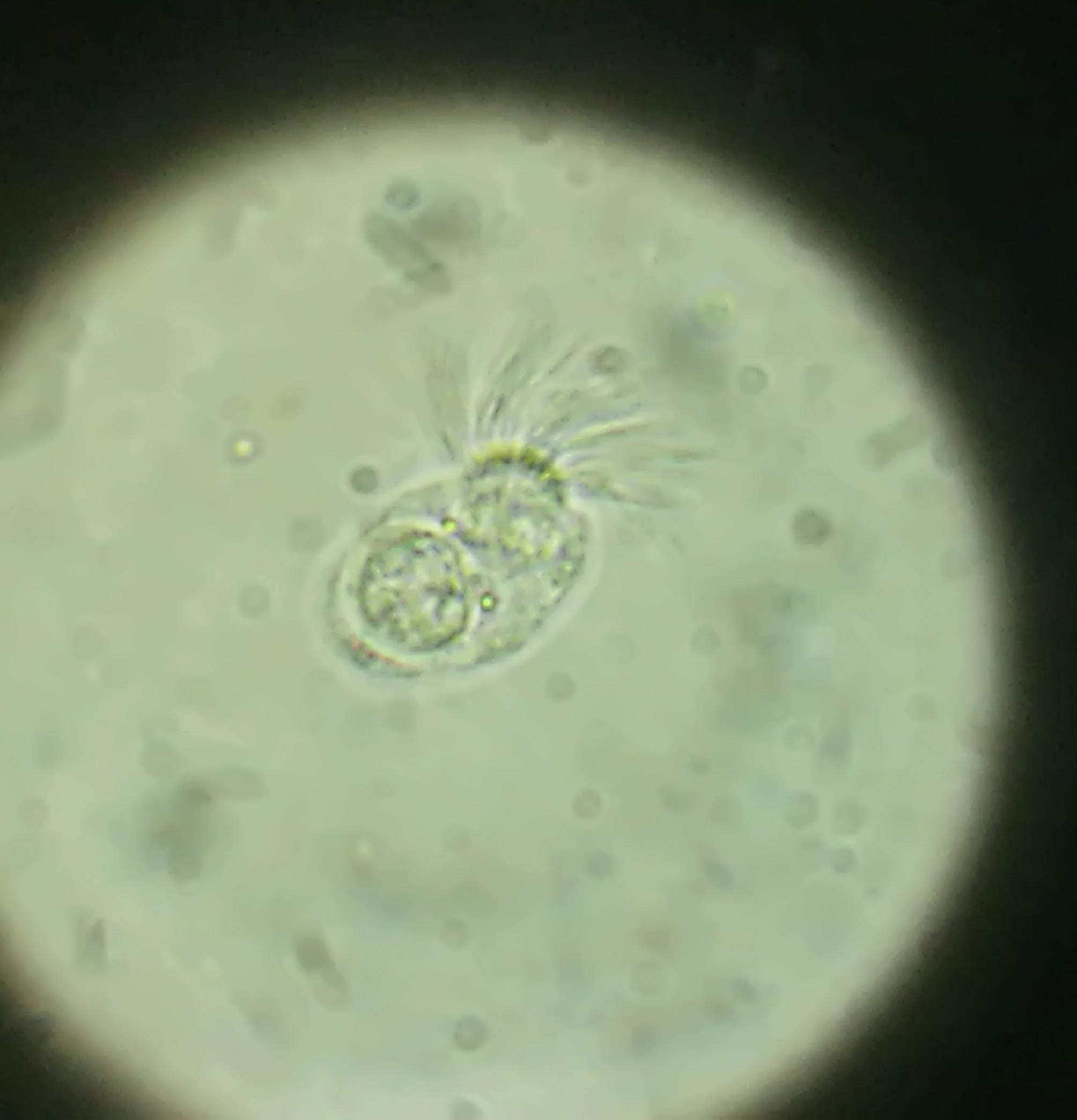
Direct smear bronchoalveolar lavage fluid specimen represent lophomonas trophozoite with irregular long flagella

**Fig. 6:**
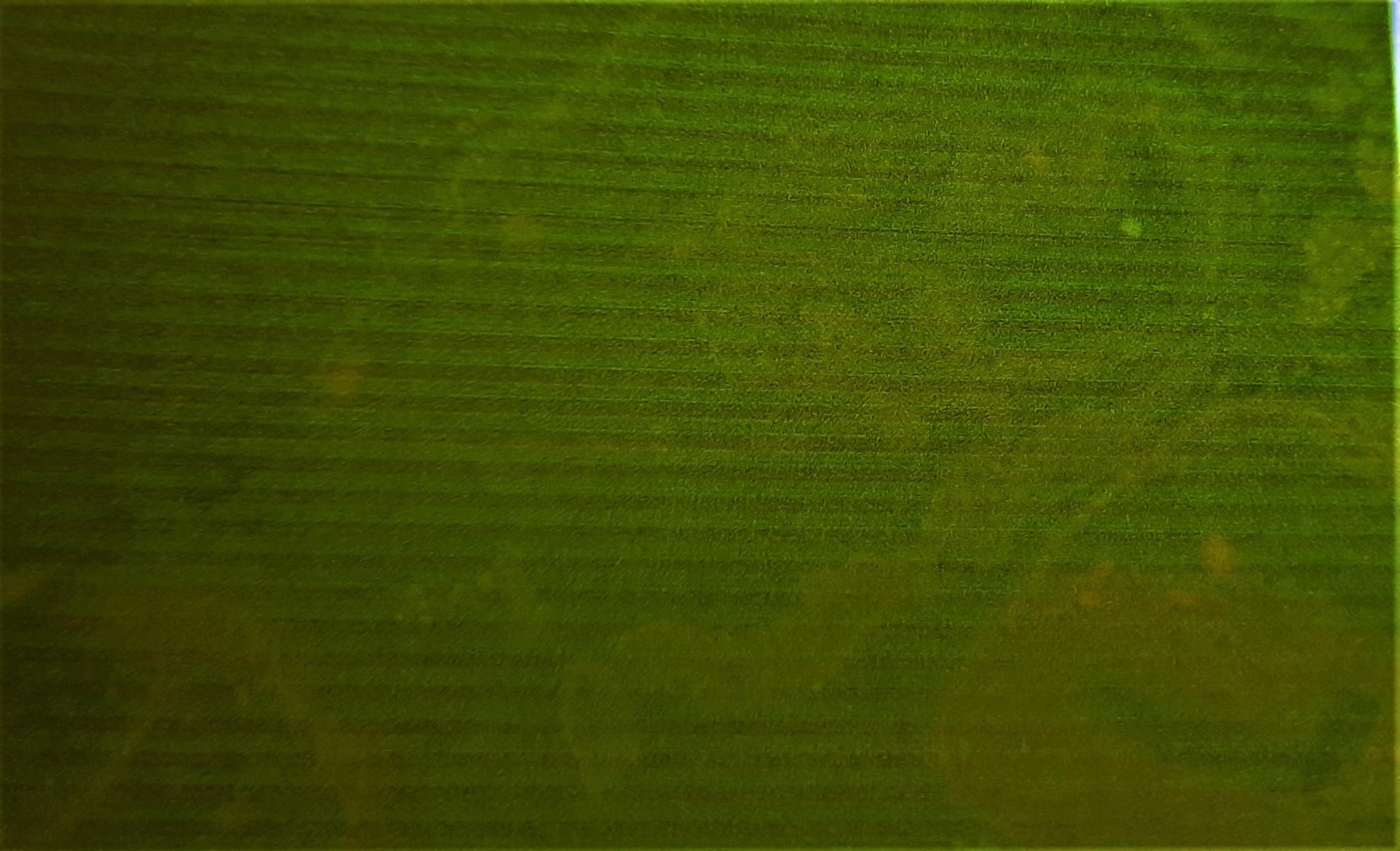
Immunofluorescence microscopic image of *Echinococcus granulosus*

Anti-Echinococcus antibodies (IgG and IgM) were detected in the patient’s serum. Fungal and bacterial infections were reported as negative in the BAL samples. The Microscopic examination of BAL revealed several live and oval flagellated lophomonas protozoa (Fig. 5), hydatid cyst protoscoleces (Fig. 6) (the larval forms of the parasites), and *M. tuberculosis*. For further evaluation of *M. tuberculosis* infection, abdominopelvic sonography was performed and it was reported normal.

Due to the positive result of the patient’s BAL and sputum culture samples, the diagnosis of co-infection of *M. tuberculosis*, *E. granulosus*, and lophomonas was confirmed. The patient was treated with metronidazole (500 mg thrice a day for two weeks), oral albendazole (400 mg twice daily for at least two weeks before the procedure and six months after surgery), and a combination of TB regimen started. The patient was discharged with oral medication prescriptions, in good general condition, and with improved clinical symptoms.

## Discussion

We admitted a 58-year-old patient with chief complaints of fever, losing weight, and cough with whitish-yellow sputum that started one month ago. Her symptoms gradually progressed, and she did not completely respond to outpatient treatment. We ordered the bronchoscopy according to abnormal laboratory tests, and CT scan findings. After further evaluation, the BAL specimen reported a negative fungal and bacterial infection in the samples. However, the microscopic examination revealed some live and oval flagellated lophomonas protozoa, hydatid cyst protoscoleces (the larval forms of the parasites), and *M. tuberculosis*. The positive anti-echinococcus antibodies were founded. Finally, the patient was treated with metronidazole, oral albendazole, and a combination *M. tuberculosis* regimen started.

Clinically significant pulmonary protozoan infections are rare but have been increasingly recognized in recent decades because of individual states of suppressed immunity. Various studies have declared the high prevalence of co-infection of *M. tuberculosis* and parasitic diseases such as *E. granulosus* and lophomonas. Furthermore, *M. tuberculosis* and parasitic diseases were the risk factors for each other. The study by Li X.,et al.2013, found that many factors possibly affect the co-infection of TB and parasitic diseases, such as socio-demographics (gender and age), underlying diseases, and living in co-endemic areas (higher prevalence of *M. tuberculosis* and parasitic infections). According to a study by Li R.,et al.2016, *L. blattarum* was considered an opportunistic infection in patients with kidney and liver allograft transplantation under corticosteroid therapy, HIV, *E. granulosus* and *M. tuberculosis* infection [1, 2, 4, 12–14].

In this study, we reported the co-infection of *M. tuberculosis*, lophomonas, and *E. granulosus*; the occurrence of these infections together is rare and has not been previously reported. In endemic areas such as Iran, the diagnosis of *E. granulosus* and *M. tuberculosis* infection can often be easily made by clinical findings, serological tests, and radiographic findings [1, 4, 7, 12].

It could be suspected that the patient first had *E. granulosus* or *M. tuberculosis* infection, and in the subsequent period, lophomonas as super infection was added to co-infections. Because lophomonas often occurs in immunocompromised patients, and *M. tuberculosis* or *E. granulosus* infection impairs the immune system, making individuals more susceptible to *﻿L. blattarum* infections and reactivating latent infections. However, considering the patient’s symptoms presented acutely and two months prior to admission, the patient’s previous routine laboratory tests and inflammatory markers were normal. In addition, due to the positive *E. granulosus*-specific IgM and IgG, active tuberculosis, our patients were more likely to have co-infection *M. tuberculosis* and *E. granulosus* with lophomonas. The study by Kalani M., et al. 2022, mentioned that the prevalence of lophomonas infection was relatively high in patients with suspected tuberculosis due to similar clinical manifestations of this co-infection (1, 2–4, 11, 12).

*L. blattarum*, *M. tuberculosis*, and *E. granulosus* infections present with untypical and non-specific clinical manifestations, such as cough, sputum, sweating, weight loss, weakness, malaise, hemoptysis, and dyspnea. These infections often involve the respiratory tract system with the most likely form of airborne transmission and are similar in terms of clinical patterns and radiographic findings. Notably, these non-specific symptoms might obscure the diagnosis and treatment, and clinical symptoms cannot distinguish these infections from other diseases. Researchers believe these infections should be considered in patients with eosinophilia, severe respiratory infection, immunosuppression, and unsuccessful antimicrobial treatment [1–14].

These infections’ X-ray and CT scan findings revealed ground-glass opacity, patchy consolidation, patchy or streaky shadows, cystic lesions, abscesses, and pleural effusion. Using these findings makes it difficult to differentiate this co-infection from other common diseases with similar radiographic findings (such as pneumonia, bronchitis, cancer, or inflammation) [1, 3, 4, 9, 12].

A bronchoscopy biopsy smear, sputum smear, or BAL can be performed to detect *L. blattarum*, tuberculosis, and hydatid in patients. However, it is challenging to differentiate *L.blattarum* and ciliated epithelial cells based on morphology under a light microscope, which can lead to misdiagnosis [2–7]. The lung epithelial cells display cilia of uniform size with consistent movement, and a terminal bar is observed at the base of the cilia. These cells also exhibit a slightly slender shape. *L. blattarum* features two tufts of flagella of varying sizes located in the anterior region, demonstrating a wavy movement. No terminal bar is observed at the base of the flagella, and the parasite is nearly round. Additionally, the morphological characteristics of this parasite were examined using an electron microscope, confirming its presence. Microscopy-based diagnosis often lacks the necessary sensitivity and specificity. Consequently, molecular-based diagnosis (PCR method) was performed in this study to identify *Lophomonas spp* [15].

As *M. tuberculosis* and *E. granulosus* are common infections in this region, and the true mechanism of lophomonas transmission has not been clearly described, the management and diagnosis of these co-infections are essential in practice. Clinicians should consider this co-infection in differential diagnoses of patients with a chronic dust allergy, unresponsiveness to antibiotic therapy, and recurrent respiratory infections.

Our study had strengths. We reported an extremely rare case of *M. tuberculosis*, *E. granulosus*, and lophomonas co-infection in a patient. In addition, according to the patient’s present illness, clinical examination, and initial radiography findings, we initially isolated the patient with the suspicion of possible opportunistic infections. We gave the necessary warnings to all the people in close contact with her and examined them for possible infections. Moreover, we prevented the patient from suffering from complications caused by each mentioned pathogen with timely diagnosis and appropriate treatment. We also faced limitations. We had to send the patient’s sample to another center for a definitive diagnosis of lophomonasis, which was time-consuming.

## Conclusion

Co-infection with tuberculosis and parasitic diseases (*E. granulosus* and lophomonas) in humans is rare and it mostly presented in co-endemic areas. Patients with risk factors such as immunodeficiency conditions or treating with immunosuppressive medications are at high risk of contracting opportunistic infections or their coexistence. Physicians should always consider the possibility of these co-infections and investigate patients with resistant symptoms.

## Data Availability

The datasets used during the current study are available from the corresponding author on reasonable request. All data generated or analysed during this study are included in this article. Further inquiries can be directed to the corresponding author.
